# *BRCA* promoter methylation in triple-negative breast cancer is preserved in xenograft models and represents a potential therapeutic marker for PARP inhibitors

**DOI:** 10.1007/s10549-024-07502-8

**Published:** 2024-10-11

**Authors:** Kavitha Däster, Jürgen Hench, Maren Diepenbruck, Katrin Volkmann, Adelin Rouchon, Marta Palafox, Jorge Gomez Miragaya, Bogdan Tiberius Preca, Christian Kurzeder, Walter Paul Weber, Mohamed Bentires-Alj, Savas Deniz Soysal, Simone Muenst

**Affiliations:** 1 Breast Center Zurich, Zurich, Switzerland; 2 University of Basel, Basel, Switzerland; 3 Institute of Medical Genetics and Pathology, University Hospital Basel, Basel, Switzerland; 4 Tumor Heterogeneity Metastasis and Resistance, Department of Biomedicine, University Hospital Basel, University of Basel, Basel, Switzerland; 5 Breast Center, University Hospital Basel, Basel, Switzerland; 6 Praxis Chirurgie Im Zentrum, Basel, Switzerland

**Keywords:** BRCA promoter methylation, Triple-negative breast cancer, PARP inhibitors, Metastatic breast cancer

## Abstract

**Purpose:**

Most triple-negative breast cancers (TNBC) are sporadic in nature and often associated with dysfunction of the *BRCA1* or *BRCA2* genes. Since somatic BRCA mutations are rare in breast cancer (BC), this dysfunction frequently is the result of *BRCA* promoter methylation. Despite the phenotypic similarities of these tumors to those with germline or somatic *BRCA* mutation, the evidence of response to PARP inhibitors is unclear.

**Methods:**

We analyzed the prevalence of *BRCA* promoter methylation in 29 BC metastases through the well-established Illumina Infinium EPIC Human Methylation Bead Chip. In cases with *BRCA* methylation, the xenograft of the same tumor was tested. Additionally, we compared BC xenografts with an identified BRCA methylation to their matched primary tumors and subsequently investigated the efficacy of PARP inhibitors on tumor organoids from a *BRCA2* promoter-methylated BC.

**Results:**

*BRCA2* promotor hypermethylation was identified in one pleural metastasis of a young patient as well as in the xenograft tissue. We also identified five more xenograft models with *BRCA2* promotor hypermethylation. Analysis of one matched primary tumor confirmed the same *BRCA2* methylation. PARP inhibitor treatment of tumor organoids derived from the *BRCA2* methylated xenograft tumor tissue of the young patient showed a significant decline in cell viability, similar to organoids with somatic *BRCA1* mutation, while having no effect on organoids with *BRCA1* wildtype.

**Conclusion:**

*BRCA* promotor hypermethylation seems to be a rare event in metastatic BC but is preserved in subsequent xenograft models and might represent an attractive therapeutic marker for PARP inhibitors.

## Background

*BRCA1* is an important breast cancer susceptibility gene first identified in 1994 [1]. Breast cells with heterozygous loss-of-function germline *BRCA* mutations can lose the remaining wild-type allele, resulting in homologous-recombination deficient DNA repair (HRD), which leads to accumulation of genetic mutations that drive breast carcinogenesis [2]. Consequently, germline *(g)BRCA1* and *BRCA2* mutations are associated with a lifetime risk of breast cancer of up to 72% [3, 4]. Typically, *BRCA2-*associated breast cancers are usually hormone receptor positive. Conversely, *BRCA1-*associated breast cancers are usually estrogen (ER) and progesterone (PR) receptor negative [4]. However, only around 15–20% of triple-negative breast cancers (TNBC) are associated with a *BRCA1* germline mutation [5, 6].

Recent studies show that about 40% of sporadic, non-familial TNBC also harbor HRD due to silencing or dysfunction of *BRCA1* or *BRCA2* [7]. Given that *BRCA1* somatic mutations are uncommon and detected in only 3–5% of sporadic TNBC, the main mechanism behind this seems to be the epigenetic inactivation of the *BRCA1* and *BRCA2* gene by aberrant addition of methyl groups in their CpG-rich regulatory regions (promoter CpG islands) [8–11]. The methylation of *BRCA* promoter results in the reduction and/or loss of the BRCA protein abundance similar to that of germline or somatic mutations [12, 13]. The available studies assessing *BRCA1* and 2 promoter methylation in breast cancer have predominantly analyzed primary breast tumors with only one recent study by Bonnet et al. showing *BRCA1* promotor hypermethylation in 4% of metastatic TNBC samples [14]. Therefore, overall, there is scarcity of studies in assessing the prevalence of *BRCA* promoter methylation in metastatic breast cancer tissue and the prevalence of BRCA 2 in this patient cohort is still yet to be determined.

The use of poly(adenosine diphosphate [ADP]–ribose) polymerases (PARP) inhibitors is an elegant therapeutic strategy with compelling antitumor activity in patients with *gBRCA* mutated tumors. Through the concept of synthetic lethality, they induce selective tumor cytotoxicity, sparing normal cells and predicting a high therapeutic index [15, 16]. This has been successfully demonstrated in phase 3 clinical trials in patients with *gBRCA* breast cancers, especially of the TNBC subtype [17, 18].

Whether therapy with PARP inhibitors can be extended to *BRCA1* promoter-methylated cancers is still under investigation. Interestingly, several recently published studies indicate that patients with advanced, *BRCA1* promoter-methylated TNBC also respond to PARP inhibitors [12, 19, 20].

The aim of our study was to assess the prevalence of *BRCA1* or *BRCA2* promotor hypermethylation in metastatic triple-negative and Luminal B breast cancer. Given that early studies show that *BRCA* methylation may confer sensitivity to PARP inhibitors, identifying *BRCA* promoter methylation in metastatic tissue (in particular TNBCs and Luminal B breast cancers) could further expand the use of this targeted therapy. Parallel to this, we aimed to investigate the response to PARP inhibitors in tumor organoids stemming from *BRCA* promoter-methylated TNBCs.

## Methods

We assessed the prevalence of *BRCA1* and *BRCA2* promoter methylation in archival metastatic biopsies as well as in TNBC xenograft tissues.

After identifying suitable biopsies in our archives, diagnoses of (metastatic) TNBC or Luminal B breast cancer were confirmed by a specialized breast pathologist (S.M.). Tumors were defined as triple-negative as follows: less than 1% of ER and PR immunoreactivity and absence of human epidermal growth factor receptor 2 (HER2) protein overexpression or gene amplification. Luminal B tumors were defined as ER-positive (> 1%), HER2-negative cancers with a proliferation rate (Ki-67) of > 25%.

The technical protocol of the methylation analysis is well-established and part of our routine diagnostic practice: for each case, depending on biopsy size, a sufficient number of sections (usually, between 2 and 6 sections of 70 μm thickness) was used for DNA isolation (Promega Maxwell FFPE kit, applicable to both native and FFPE tissue). After bisulfite-conversion and low-level amplification, the DNA was hybridized to Illumina Infinium Human Methylation Beadchips (EPIC V1, 850 K), and the array read on an Illumina scanner (service provided by the LIFE&BRAIN research platform, Bonn/Germany). The resulting data (IDAT format) were then preprocessed and normalized (SWAN), mapped to the genome and converted into beta values (which represent methylation state at each scanned site; all pre-processing via minfi) [21, 22]. The EPIC 850 K array was used, and the following cg IDs were analyzed for BRCA 1 and 2.

- BRCA1; cg09698981, cg09698981, cg05153735, cg05153735, cg27253386, cg27253386, cg08157964, cg08157964, cg09102017, cg09102017, cg15865175, cg15865175, cg25497877.
- BRCA2; cg16029534, cg16029534, cg16919093, cg16919093, cg07054526, cg07054526, cg19088651, cg19088651, cg08386886, cg08386886, cg08993267, cg08993267, cg24806953, cg24806953, cg20187250, cg20187250, cg15419295, cg15419295, cg16963062, cg16963062, cg16630982, cg16630982, cg21253966, cg21253966, cg04110421, cg04110421, cg04658354, cg04658354, cg17301289, cg17301289, cg09441966, cg09441966, cg20760063, cg20760063, cg10893007, cg10893007, cg12182452, cg12182452, cg09831010, cg09831010, cg25288140, cg25288140, cg15065591, cg15065591, cg20185525, cg20185525, cg26879546.

This study was approved by the Ethikkommission Nordwest- und Zentralschweiz (EKNZ, Proposal Number 2014-397).

### Breast cancer patient-derived xenografts (PDX) models

Human breast cancer tissues were from the University Hospital Basel after obtaining written informed consent from the patient (Study ID 2018-00729 approved by the Swiss authorities (EKNZ)). PDX models were established as previously described [23]. Briefly, patient breast tumor tissues were surgically resected and directly transplanted into the mammary fat pad of female NSG (NOD-scid-Il2rg^null^) mice.

### PDX-derived breast cancer tumor organoid model

The breast cancer organoid model, UHB150X_O, has been derived from the mammary tumor of the PDX model UHB150X (mouse passage one). Briefly, the tumor tissue was chopped and then enzymatically digested with liberase (Roche #05466202001) and DNase I (Stemcell #07900) for 1 h at 37C. The cell suspension was passed through a 100 μm and a 40 μm cell strainer and washed with PBS. Murine cells were removed with the Mouse Cell Depletion kit (Miltenyi Biotec #130-104-694) using a magnetic separator. The human identity of the tumor organoids was verified by a CD298 + staining (BioLegend #341,06), a beta-subunit of the Na + /K + ATPases specific for human cells [24]. PDX-derived human breast cancer cells were cultured as tumor organoids in Matrigel (Corning #356231) in 24-well ULA plates, at 37 C with 5% O_2_ and using the culture medium published by Sachs et al. [25]. For organoid passaging, organoids in Matrigel were collected and incubated 1:1 with dispase (Gibco #17105-041) for 60 min at 37 °C. After Matrigel digestion, tumor organoids were washed with PBS, resuspended in accutase (Sigma, #A6964) and incubated for 5–7 min at 37 °C. After disaggregation, single cells were washed with PBS twice and reseeded in Matrigel or used for PARP inhibitor treatment.

### PARP inhibitor treatment of UHB150X_O

PDX-derived human tumor organoids were prepared as single cells as described above and were plated in 30% Matrigel as technical triplicates in a 384-well plate (Greiner #781091) and cultured at 37C, 5% O_2_ with the described culture medium (NAC and ROCKi were removed from the medium) [25]. The TNBC cell lines MDA-MB-231 (BRCA wt) and MDA-MB-436 (BRCA1 mut) served as cellular treatment controls [26]. After 1 day of 3D culture, cell lines and PDX-derived tumor organoids were treated with Olaparib and Rucaparib (Selleckchem #S1060, #S4948) for 6 days. The treatment was refreshed once during the treatment time. Afterwards, tumor organoids were fixed with 4% PFA for 30 min at RT and permeabilized with 0.3% Triton-X100 in PBS for 20 min. Nuclei of cells were stained with DAPI (ThermoFisher #D1306) and tumor organoids were imaged using the CQ1 spinning disk confocal microscope (Yokogawa). Cell nuclei were quantified with the integrated CQ1 software. The experiment was repeated two times (*n* = 1–2), and the results were integrated. Statistical analysis and generation of graphs were performed using the Prism software (GraphPad Software, version 9.3.1.). Data are shown as mean ± standard deviation (SD).

## Results

From the database and the archives of the Institute of Pathology, a total of 31 patients were identified. Two patients were excluded as one patient had only a phylloides tumor and the other patient had a HER2-positive cancer. Therefore, a total of 29 patients with metastatic Luminal B or TNBC were identified. The mean age was 62 years (range 28–86 years). 4 (14%) patients had a TNBC, and 25 (86%) were of Luminal B subtype. Metastatic sites included bone 14 (48%), lung 9 (31%), liver 5 (17%) and brain 1 (4%).

Additionally, tissues from 24 breast cancer xenograft models were included in the study. Seventeen of these xenografts were from TNBC, 6 from Luminal B cancers, and one was initially taken from a HER2-positive cancer.

**Fig. 1:**
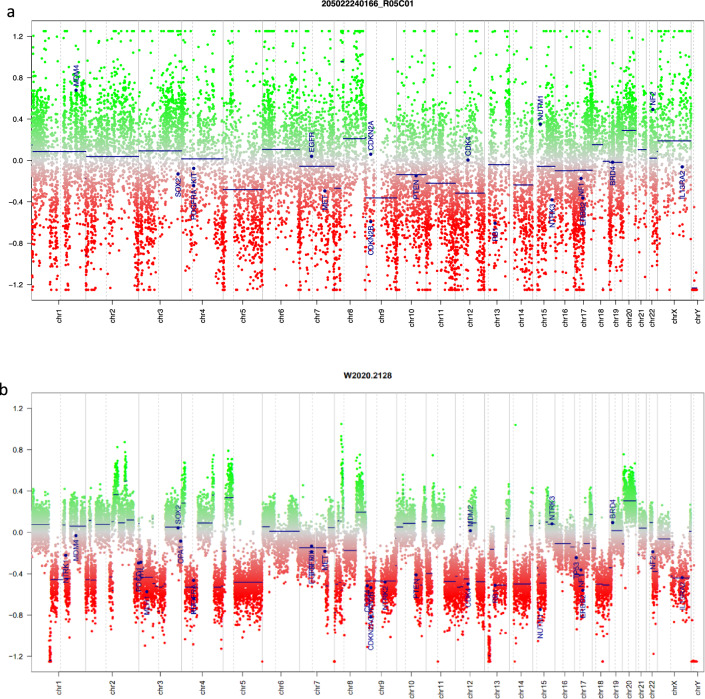
**a** CNV aberrations from the pleural metastasis of patient 1. **b** The xenograft of the same tumor essentially retains the CNV aberrations from the original tumor. Both figures have a large number of shorter-range alterations, reflecting the effect of HRD

Out of the 29 metastatic tissues assessed, we were unable to assess methylation in 9 due to DNA degradation. Among the rest of the 20 specimens, we identified one pleural metastasis from a TNBC with a distinct *BRCA2* methylation (5%). The associated copy number variant (CNV) profile showed a high amplitude, compatible with a homologous recombination defective (HRD) tumor (Fig. 1). Importantly, we also detected the same *BRCA2* methylation in the tissue from the associated xenograft model, proving that this methylation was conserved through the generation of the PDX (Fig. 1). Furthermore, *BRCA2* methylations were present in 5 additional TNBC xenografts. For one of these xenografts, the primary tumor was available, and harbored an identical *BRCA2* methylation. Unfortunately, the primary tumors of the remaining 4 *BRCA2* methylated xenografts were not available and thus could not be tested.

The patient with the detected *BRCA2* methylation in the pleural metastasis as well as in the associated xenograft was diagnosed with a triple-negative breast carcinoma in 2016 at the age of 25. The patient underwent neoadjuvant chemotherapy with epirubicin, cyclophosphamide, paclitaxel and carboplatin, followed by wide local excision with sentinel lymph node biopsy a postoperative radiotherapy. She presented with a non-invasive local recurrence (ductal carcinoma in situ) 11 months later, which was treated with skin-sparing mastectomy and a Deep Inferior Epigastric Artery Perforator (DIEP) reconstruction. Over the following months, she had two more local recurrences at the mastectomy site, which were both again surgically excised, followed by second- and third-line chemotherapy. Unfortunately, she developed liver, lung and brain metastases for which she received multiple palliative lines of chemotherapy including Mitoxanthron, Methotrexate and Mitomycin. Importantly, she received genetic testing, and no germline mutations in *BRCA1* or *BRCA2* could be detected, so she was never treated with PARP inhibitors. Unfortunately, by January 2020 she suffered from significant disease progression, and she subsequently passed away, four years after the initial diagnosis, at the age of 29.

Next-generation sequencing analysis of the locally recurrent tumor as well as the pleural metastasis revealed a *BRCA2* mutation in exon 11 (p.N1279S), which was somatic, given that germline testing for *BRCA1* and *BRCA2* revealed no mutations in either of these genes. *BRCA2* exon 11 (~ 65% of the coding sequence) is a coldspot, where missense variants are very unlikely to be pathogenic [27].

This alteration in the sequence results in the replacement of asparagine, which is a neutral and polar amino acid, with serine, another neutral and polar amino acid, at codon 1279 of the *BRCA2* protein (p.Asn1279Ser). This specific genetic variant is not found in population databases (absent in gnomAD). It has been observed in individuals diagnosed with breast and/or ovarian cancer, as well as in those unaffected by these conditions [28]. Advanced computational analysis of the protein sequence and its biophysical characteristics, including structural, functional, and spatial data, amino acid conservation, physicochemical variability, residue mobility, and thermodynamic stability, suggests that this missense variant is unlikely to disrupt the function of the *BRCA2* protein. It is thus considered benign or likely benign [27]**.**

The second patient with the *BRCA2* methylation in her primary tumor as well as the associated xenograft tissue is also a young patient who was diagnosed with a TNBC at the age of 29, during her second pregnancy. Despite a large tumor size of 45 mm, she opted for primary surgery and underwent a mastectomy with sentinel node biopsy, followed by four cycles of adjuvant chemotherapy with epirubicin and cyclophosphamide, as well as carboplatin and taxol. She subsequently gave birth to a healthy girl and is currently free of disease, 3 years after the initial diagnosis.

**Fig. 2:**
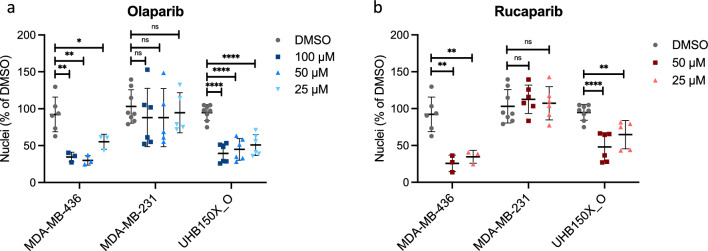
Tumor organoids cultured ex vivo in Matrigel from the *BRCA2* methylated TNBC PDX tumor tissue (UHB150X_O). Graphs display the responses (nuclei counts in percent normalized to DMSO control treatment) to treatment with Olaparib and Rucaparib for 6 days. MDA-MB-436 (BRCA1 mutated) and MDA-MB-231 (BRCA wt) served as treatment controls. *N* = 1–2 with 3–4 technical replicates each. Shown are mean ± SD and statistical significance (ns: not significant; * < 0.05; **: < 0.01, ****: < 0.0001; unpaired *t*-test)

Simultaneously, tumor organoids derived from the PDX model from the first patient with pleural TNBC metastasis with *BRCA2* methylation were treated with two different PARP inhibitors (Olaparib and Rucaparib) in three different concentrations (25, 50 and 100 µM). Two breast cancer cell lines cultured in matrigel served as cellular controls. The TNBC cell line MDA-MB-436 display a *BRCA1* mutation, the TNBC cell lines MDA-MB-231 displays a *BRCA* wildtype status. After PARPi treatment for 6 days, the tumor organoids from the xenograft models of the *BRCA2* hypermethylated patient showed a decline in tumor cell nuclei, in contrast to the organoids with BRCA wildtype, but similar to the *BRCA1* mutated organoids (Fig. 2). These results further support the notion that *BRCA2* methylation leads to a similar phenotype as a *BRCA* mutation and that tumors with inactivation of *BRCA* through methylation may thus also benefit from PARP inhibitor treatments. Unfortunately, the patient had already succumbed to her disease by the time these experiments were conducted on the organoids from her tumor tissue.

## Discussion

We find that *BRCA* methylation seems to be a rare event in metastatic TNBC and luminal B breast cancers. However, if present, these tumors show a CNV profile compatible with HRD. Importantly, our reported incidence may be an underestimation, since we also found *BRCA2* methylations in 6 out of 24 TNBC xenografts of which unfortunately, we did only have the primary tumor tissue to analyze for one case. This case indeed also showed a *BRCA2* methylation in the corresponding primary tumor, which suggests that this alteration is generally preserved during PDX generation. Including the *BRCA* methylations found in the xenografts, the incidence rises to 7 out of 21 TNBC cases, or 33.3%. The prevalence of *BRCA1* promoter methylation in sporadic breast cancer is variable, depending on the selected cohort, in particular the molecular subtype [29, 30]. Stefansson et al. showed that among 965 sporadic breast cancers, 3% demonstrated *BRCA1* promoter methylation, however, 91% of these tumors were ER negative and 67% were basal like [31]. The increased prevalence of *BRCA1* promoter methylation in TNBC was subsequently demonstrated in other studies reporting rates as high as 59% [11, 12, 30, 32–35]. Only one study assessed the prevalence of *BRCA2* methylation in sporadic breast cancers (all molecular subtypes) showing a prevalence of 12.5% [36].

In our study, no Luminal B cancers harbored a *BRCA* methylation, however, this could be due to the limited number of patients included in this study. The published literature however does reflect our findings. Jacot et al. assessed 155 patients with sporadic breast cancers which were matched for TNM status and found *BRCA1* promoter methylation in 29% of TNBC and only in 5 and 2% of ER-positive and HER2-positive breast cancers respectively [30]. Additional studies have also demonstrated lower prevalence of *BRCA1* promoter methylation among ER-positive breast cancers [29–31]. Importantly, the prevalence of *BRCA1* promoter methylation in Luminal B breast cancers has been reported to be higher compared to the Luminal A subtypes. Grushko et al. reported the presence of *BRCA1* promoter methylation of up to 30% in Luminal B breast cancers when compared to luminal A and HER2-positive cancers (18 and 4% respectively) [29].

*BRCA* methylation has been shown to add prognostic value in TNBC. *BRCA1* methylation is associated with younger age, higher grade, mitotic count scores and Ki-67 index, and the presence of lymphovascular invasion [13, 30, 32, 33]. Studies have also demonstrated that *BRCA1* promoter methylation is a possible marker of poor disease outcome, as it is associated with reduced disease-free survival (DFS), overall survival (OS) and an increased risk of breast cancer-specific mortality, and also seems to be associated with increased sensitivity to adjuvant chemotherapy [13, 34, 35, 37, 38]. The aforementioned study investigating *BRCA2* methylation in sporadic breast cancers showed that it is associated with more grade 3 carcinomas, however so far there are no reports on prognostic impact of *BRCA2* methylations in breast cancer [36]. Our findings suggest that *BRCA2* methylation may also be a negative prognostic factor, since the two patients with *BRCA2* methylation we studied were both very young, with highly aggressive TBNC tumors.

PARP inhibitors have become increasingly important in the treatment of *gBRCA* mutated breast cancer, entering first-line therapy. Clinical trials have indicated superior progression-free survival (PFS) with PARP inhibitor monotherapy compared with chemotherapy for *gBRCA* muted, HER2-negative metastatic breast cancer, leading to the approval of the PARP inhibitors Olaparib and Talazoparib for this patient population [17, 39].

As *BRCA*-methylated breast cancers seem to be phenotypically similar to tumors with somatic or germline *BRCA* mutations, these patients may also benefit from PARP inhibitors, especially in a metastatic setting. Veeck et al. demonstrated that *BRCA1* hypermethylation in breast cancer cell lines conferred the same degree of sensitivity to three PARP inhibitors as *BRCA1* mutation [12]. Similarly, Cai et al., also using human breast cancer cell lines, showed that *BRCA1* hypermethylation conferred a sensitivity to Olaparib similar to that of *BRCA1* mutated cells. Olaparib did not only inhibit the growth of cells in a concentration and time dependent manner, but also reduced *BRCA*1 promoter methylation level [19].

Importantly, Kawachi et al. recently performed a post hoc analysis of *BRCA1* methylation in samples obtained from patients with advanced and metastatic TNBC from a phase I/II trial of Olaparib in combination with Eribulin [20]. The results were promising in that patients with high *BRCA1* promoter methylation showed better 6-month PFS compared with patients without *BRCA1* methylation when treated with Olaparib. However, this study had several limitations, including the small number of patients, and that the origin of the biopsy specimens was not mentioned. Therefore, the study could not definitely conclude that *BRCA1* methylation reliably predicts the response to PARP inhibitors similar to that of *gBRCA* mutated cancers [20].

Concerning our two breast cancer patients with a proven *BRCA2* methylation in the cancer cells, the use of PARP inhibitors may very well have been an option for the first patient in the metastatic setting. Unfortunately, due to negative genetic testing, she did not receive this line of therapy. The second patient is currently alive and well, however, the possibility of PARP inhibitor therapy should be kept in mind if she were to progress.

Adding methylation analysis into the diagnostic workup of TNBC metastases is a cost-effective method to identify additional patients with HRD breast cancers in cases where somatic or germline genetic *BRCA* testing is negative. Especially in widely metastasized patients, identifying an HRD in the tumor could open up additional therapeutic possibilities with well-established drugs.

Interestingly, our studies shows that *BRCA* methylation seems to be conserved through the metastasic process and even when establishing the PDX, a result which has been obtained for the first time for TNBC. Therefore, if the primary tumor is not available, then testing the metastatic tissue or or the PDX would also be possible in order to identify patients who may benefit from PARP inhibitor treatment [40].

## Limitations

The number of metastatic breast cancer patients included in this study is certainly low (*n* = 28). Additionally, we did not have the primary tumor of one of the patients with *BRCA2* methylation for comparison, so we are unable to say if this methylation is something that occurred during the metastatic progression of the cancer. However, since most patients succumb to their metastatic disease, detection of a *BRCA* methylation in the metastatic tissue would be clinically more relevant. Additionally, it is not possible to say with absolute certainty if the detected coldspot mutation of the BRCA2 gene in the locally recurrent tumor of the young patient with proven BRCA2 methylation, from where the organoids were created led to an additional susceptibility to PARP inhibitors, even though structural analysis suggest that it does not disrupt the function of the *BRCA2* protein and should thus not lead to a HRD.

## Conclusion

*BRCA* methylation is a rare event in metastatic TNBC but seems to lead to HRD comparable to tumors with germline or somatic *BRCA* mutations. Our study is the first to report the occurrence of *BRCA2* methylation in metastatic breast cancer. Our results also show that *BRCA* methylation is conserved through the metastatic process and in the PDX and may be an additional therapeutic marker for using PARP inhibitors.

## Data Availability

The datasets generated during and/or analyzed during the current study are not publicly available but are available from the corresponding author on reasonable request.
